# Genome‐wide association and replication studies for handedness in a Korean community‐based cohort

**DOI:** 10.1002/brb3.3121

**Published:** 2023-06-20

**Authors:** Youhyun Song, Dasom Lee, Ja‐Eun Choi, Ji Won Lee, Kyung‐Won Hong

**Affiliations:** ^1^ Department of Family Medicine Gangnam Severance Hospital Yonsei University College of Medicine Seoul South Korea; ^2^ Healthcare Research Team, Health Promotion Center Gangnam Severance Hospital Yonsei University College of Medicine Seoul South Korea; ^3^ Theragen Bio Co. Ltd. Gyeonggi‐do South Korea; ^4^ Department of Family Medicine Severance Hospital Yonsei University College of Medicine Seoul South Korea; ^5^ Institute for Innovation in Digital Healthcare Yonsei University Seoul South Korea

**Keywords:** ambidexterity, genome‐wide association study, genetic, handedness, left‐handedness

## Abstract

**Introduction:**

Handedness is a conspicuous characteristic in human behavior, with a worldwide proportion of approximately 90% of people preferring to use the right hand for many tasks. In the Korean population, the proportion of left‐handedness is relatively low at approximately 7%–10%, similar to that in other East‐Asian cultures in which the use of the left hand for writing and other public activities has historically been oppressed.

**Methods:**

In this study, we conducted two genome‐wide association studies (GWASs) between right‐handedness and left‐handedness, and between right‐handedness and ambidexterity using logistic regression analyses using a Korean community‐based cohort. We also performed association analyses with previously reported variants and our findings.

**Results:**

A total of 8806 participants were included for analysis, and the results identified 28 left‐handedness‐associated and 15 ambidexterity‐associated loci; of these, two left‐handedness loci (*NEIL3* [*rs11726465*] and *SVOPL* [*rs117495448*]) and one ambidexterity locus (*PDE8B/WDR41* [*rs118077080*]) showed near genome‐wide significance. Association analyses with previously reported variants replicated *ANKS1B* (*rs7132513*) in left‐handedness and *ANKIB1* (*rs2040498*) in ambidexterity.

**Conclusion:**

The variants and positional candidate genes identified and replicated in this study were largely associated with brain development, cerebral asymmetry, neurological processes, and neuropsychiatric diseases in line with previous findings. As the first East‐Asian GWAS related to handedness, these results may provide an intriguing reference for further human neurologic research in the future.

## Graphical abstract

In this GWAS regarding handedness, 8806 Korean subjects were included for analysis and the results identified 28 left‐handedness‐associated and 15 ambidexterity‐associated loci; of these two left‐handedness loci [NEIL3 (rs11726465) and SVOPL (rs117495448)] and one ambidexterity locus [PDE8B/WDR41 (rs118077080)] showed genome‐wide significance. We also performed association analyses with previously reported variants in Caucasians and replicated ANKS1B (rs7132513) in left‐handedness and ANKIB1 (rs2040498) in ambidexterity.

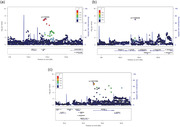

## 1 INTRODUCTION

Handedness is a prominent variation in human behavior. There are various hypotheses based on extensive studies regarding the development of handedness, and these remain highly controversial. However, many studies regard neurological or brain asymmetry as the main cause (Kang et al., 2017; Papadatou‐Pastou et al., 2020; Paracchini, 2021). A recent genome‐wide association study (GWAS) meta‐analysis regarding handedness based on Caucasians, the largest to date, reported 41 genes significant for left‐handedness and seven genes significant for ambidexterity (Cuellar‐Partida et al., 2021). According to this study, the list of genome‐wide significant associations includes multiple variants associated with genes involved in microtubules, brain morphology, and neuropsychiatric traits.

Globally, the proportion of left‐handed people is approximately 10%−12%; in Korean adults, the proportion of left‐handedness is 7%−10% (Hardyck & Petrinovich, 1977). The probability of being left‐handed may be affected by the time of birth or geographical region and may have a large cultural influence, along with genetic pathways. In the past, writing or performing public acts using one's left hand has been strictly forbidden in several East‐Asian cultures, often accompanying forced “correction” to right‐handedness (Zheng et al., 2020). Accordingly, based on a 2013 “Gallup Korea” survey, the proportion of left‐handed Koreans in their 20s was the highest at 8%, and that among those in their 60s was the lowest at 2% (Gallup (Korea),^2013^). Under such circumstances, genetic factors can be considered to play a strong role in growing up to be left‐handed despite cultural restraints in the elderly Korean population.

To the best of our knowledge, there have been no GWAS analyses on handedness that are focused on East‐Asians. In this study, we aimed to identify the genetic variants associated with handedness in an East‐Asian population‐based cohort. We also attempted to examine previously reported genetic variants regarding handedness using our results from Korean data.

## 2 MATERIALS AND METHODS

### 2.1 Study population

This study used data from the KoGES—Ansan and Ansung conducted by the Korea Centers for Disease Control and Prevention. The KoGES aims to investigate the genetic and environmental etiology of noncommunicable chronic diseases such as type 2 diabetes, obesity, and cardiovascular diseases. In total, 8806 participants between the ages of 40 and 69 years were recruited from 2001 to 2002 through two population‐based prospective cohort studies conducted in the Ansung (*n* = 4178) and Ansan (*n* = 4628) regions of South Korea. After excluding participants with missing values (*n* = 34), the number of participants included in this study totaled 8806. Detailed information about the KoGES has been described in a previous report (Kim & Han, 2017), including identity‐by‐state, multidimensional scaling, and principal component analyses (Cho et al., 2009). This research project was approved by the Institutional Review Board of Theragen Etex (approval numbers: 700062‐20190819‐GP‐006‐02). This study complied with the ethical principles of the Declaration of Helsinki. Baseline and follow‐up recruitment and assessment were conducted after obtaining written informed consent.

### 2.2 Data collection

The study participants were interviewed by trained medical staff regarding their lifestyle, sociodemographic status, disease history, and other factors. Handedness was classified into three categories by questionnaire (right‐handedness, left‐handedness, and ambidexterity), and trained staff completed the questionnaire by a direct interview. Anthropometric and clinical measurements were also obtained through standard protocol to evaluate health status, as detailed previously (Kim & Han, 2017).

### 2.3 Genotypes

Genotype data were provided by the Center for Genome Science, Korea National Institute of Health. The genotype data were generated by the Korea Biobank Array (Affymetrix, Santa Clara, CA, USA) (Moon et al., 2019). The experimental results were filtered using the following quality control criteria: call rate >97%, minor allele frequency >1%, and Hardy–Weinberg equilibrium test *p*‐value < 1 × 10^−5^. After the quality control procedures, experimentally determined genotypes were used as the genotype imputation dataset against the 1000 Genome Phase 1 and 2 Asian panels. Finally, the GWAS included 7,975,321 single nucleotide polymorphisms (SNPs) from chromosomes 1 to 22.

### 2.4 Statistical analyses

The proportions of handedness were compared using chi‐squared tests for the three handedness categories (right‐handedness, left‐handedness, and ambidexterity). Two GWASs examined the likelihood of left‐handedness or ambidexterity rather than right‐handedness. The GWASs were performed via logistic regression, adjusting for age, sex, and residence area as covariates, using PLINK version 1.09. The genomic inflation factor lambda was 1.00 for both GWASs (see the quantile‐quantile plot in Figure S1). Odds ratios (ORs) and 95% confidence intervals (95% CIs) were calculated. The threshold for significant associations was defined as *p*‐value < 5.00 × 10^−8^. The top significant SNPs obtained through these analyses were classified into loci based on whether they satisfied the criteria of *D*' > 0.8 and *R* ^2^ > 0.8; the LD link tool (https://ldlink.nci.nih.gov/?tab=home) was used to confirm the linkage disequilibrium with the cluster SNPs at a range of ± 10 kb.

### 2.5 Replication test

To understand the similarity of the handedness‐related genetic backgrounds between Korean and European populations, we also examined previously reported handedness‐related SNPs in our dataset.

## 3 RESULTS

### 3.1 Population characteristics

Handedness data were obtained from self‐reported questionnaires. Among 8806 individuals aged 40−69 years (mean age, 52.2 ± 8.9 years), 4167 (47.3%) were male. Further, 7722, 340, and 744 identified themselves as right‐handed, left‐handed, and ambidextrous, respectively. Females showed a higher tendency for right‐handedness than males (53.2% females vs. 46.8% males), whereas males showed more left‐handedness than females (52.6% males vs. 47.4% females). No significant difference between sexes was found with ambidexterity (50.0%) (Table 1).

**TABLE 1:** Baseline demographic of the study population.

		Handedness groups			
Variables	Total	Right‐handedness	Left‐handedness	Ambidexterity	*p*‐Value
*N* (%)	8806	7722 (87.69)	340 (3.86)	744 (8.45)	
Age (mean ± SD)	52.2 ± 8.9	52.3 ± 9.0	51.9 ± 8.8	51.3 ± 8.5	9.15E‐03
Sex (male %)	4167 (47.3)	3616 (46.8)	179 (52.6)	372 (50.0)	3.39E‐02
Residence area (Ansung/Ansan %)	47.4/52.6	46.8/53.2	56.8/43.2	49.7/50.3	6.61E‐04

### 3.2 Replication results

Cuellar‐Partida et al. identified 41 left‐handedness loci and seven ambidexterity loci in people of European ancestry. They also found a low genetic correlation between the two traits (*Rg* = 0.26). We investigated the associations of the 48 handedness loci identified by Cuellar‐Partida et al. and the 43 loci identified in the Korean population in this study (*p*‐value < .05). *ANKS1B* (*rs7132513*, OR = 0.67, 95% C.I. = 0.45−0.99, *p*‐value = .04831) in left‐handedness and *ANKIB1* (*rs2040498*, OR = 0.77, 95% C.I. = 0.64−0.93, *p*‐value = .006035) in ambidexterity showed associative significance. The reported association between *ANKIB1* and ambidexterity was greater than 1, but Cuellar‐Partida et al. expressed this based on the effective allele rather than the minor allele. In this study, our result was smaller than 1 based on the minor allele, which can be considered an equally reproducible result (Table 2, see Table S3 for the original GWAS replication results). In addition, we performed a meta‐analysis with the results from the 23andMe dataset included in Cuellar‐Partida et al.’s study. The two replicated variants showed increased statistical significance in the meta‐analysis (see Table S4).

**TABLE 2:** Significant single nucleotide polymorphisms (SNPs) in the replication genome‐wide association study (GWAS).

							Logistic regression analysis				HWE
rs ID	Chromosome	Base pair	Proximal gene	Minor allele frequency	Minor allele	Major allele	Odds ratio	95% Confidence interval	*p*‐Values	Sample size^a^	*p*‐Values
Left‐handedness											
rs7132513	12	100324975	*ANKS1B*	0.06	G	C	0.67	0.45–0.99	4.83E‐02	1107	8.40E‐01
Ambidexterity											
rs2040498	7	91899117	*ANKIB1*	0.11	T	A	0.77	0.64–0.93	6.04E‐03	1282	5.98E‐01

Abbreviations: HWE, Hardy‐Weinberg equilibrium.

^a^ Sample size for 80% power at *α* = .05 is based on the parameter of this population, including minor allele frequency and effect size of the markers.

### 3.3 GWAS findings

**FIGURE 1:**
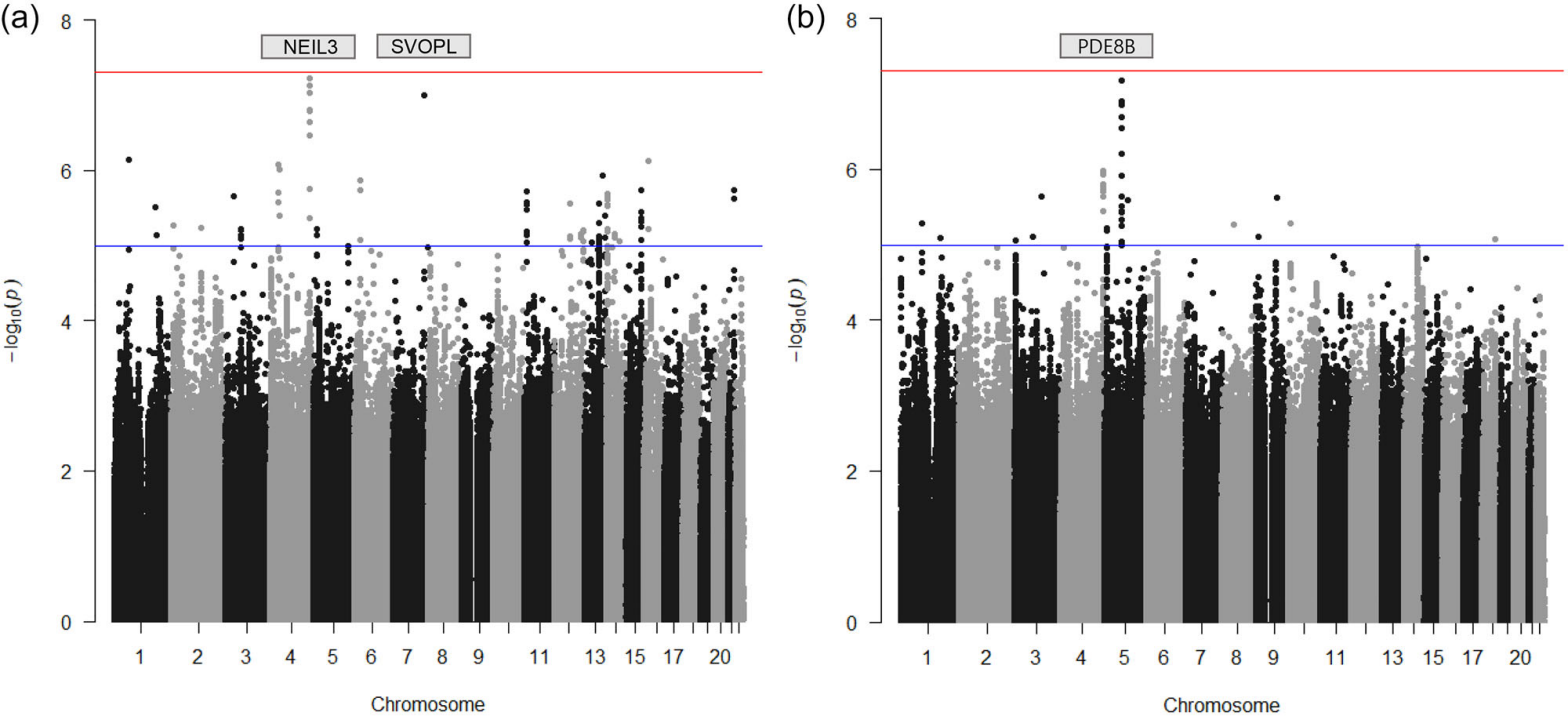
Manhattan plots showing the genome‐wide association study (GWAS) results. (a) Left‐handedness and (b) ambidexterity. Genome‐wide significant *p*‐value criteria (*p*‐value < 5 × 10^−8^) are shown in red, and genome‐wide suggestive *p*‐value criteria (5 × 10^−8^ ≤ *p*‐value < 1 × 10^−5^) are shown in blue. The *y*‐axis is –log_10_ (*p*‐value) of the single nucleotide polymorphisms (SNPs), and the *x*‐axis is chromosomal position (hg19). Summary statistics for the lead variants at genome‐wide significant loci are presented in Table 3 along with the gene nearest to the lead SNP. Candidate genes were identified as the gene nearest to the lead SNPs with nearby variant clusters showing similar significance.

We identified 28 and 15 loci related to left‐handedness and ambidexterity, respectively, which showed genome‐wide suggestive significance (*p*‐value < 10^−5^); the relevant GWAS results are presented in Table 3 and shown as Manhattan plots in Figure 1. There were no common loci between the two traits. From the 28 loci related to left‐handedness, 20 candidate genes were identified by proximity (*NEIL3* [*rs11726465*], *SVOPHL* [*rs117495448*], *SGIP1* [*rs78509271*], *KLB* [*rs28573932*], *CLYBL* [*rs77395534*], *NTRK3* [*rs56069748*], *RIPK4* [*rs3746895*], *CX3CR1* [*rs938207*], *MYRFL* [*rs192721285*], *CACNA1E* [*rs3766982*], *ARHGEF7* [*rs35337719*], *TRIB2* [*rs142891714*], *CDH18* [*rs770303*], *TAFA1* [*rs907082*], *TMEM132D* [*rs1451902*], *RNFT2* [*rs140649861*], *PELI2* [*rs76895919*], *PLA2G4A* [*rs77239173*], *CIT* [*rs74436390*], and *INTS6* [*rs142002811*]), and from 15 loci related to ambidexterity, 13 candidate genes were identified (*PDE8B/WDR41* [*rs118077080*], *IGSF11* [*rs138907675*], *SYK* [*rs182637278*], *ST8SIA4* [*rs79041957*], *WDR41* [*rs145242715*], *GATA3* [*rs263422*], *GLMN* [*rs150150088*], *SNAI2* [*rs80114285*], *CTNND2* [*rs4476693*], *ROBO1* [*rs75578608*], *PTPRD* [*rs7862596*], *ST8SIA3* [*rs12606549*], and *TRNT1* [*rs186743*]) (the original GWAS results in their entirety are shown in Table S1 [left‐handedness] and Table S2 [ambidexterity].)

**TABLE 3:** Single nucleotide polymorphisms (SNPs) associated with handedness by genome‐wide association study (GWAS).

							Logistic regression analysis				HWE
rs ID	Chr	Base pair	Proximal gene	Minor allele frequency	Minor allele	Major allele	Odds ratio	95% Confidence interval	*p*‐Values	Sample size^a^	*p*‐Values
Left‐handedness											
rs78509271	1	67194629	*SGIP1*	0.06	T	C	2.00	1.50–2.54	7.19E‐07	245	2.78E‐01
rs3766982	1	181646981	*CACNA1E*	0.02	G	A	2.70	1.78–4.08	3.05E‐06	359	1.78E‐01
rs77239173	1	186736169	*PLA2G4A*	0.02	T	C	2.30	1.60–3.33	7.39E‐06	379	1.00E+00
rs142891714	2	12739030	*TRIB2*	0.01	T	C	2.80	1.81–4.44	5.32E‐06	398	4.13E‐01
rs113690115	2	137242045	2q22.1	0.04	C	T	2.00	1.50–2.79	5.90E‐06	379	1.00E+00
rs938207	3	39296828	CX3CR1	0.08	A	G	1.70	1.38–2.19	2.21E‐06	314	6.75E‐01
rs907082	3	68523156	*TAFA1*	0.08	A	G	1.80	1.37–2.23	6.07E‐06	271	1.14E‐01
rs28573932	4	39445596	*KLB*	0.01	T	G	3.30	2.05–5.27	8.49E‐07	381	2.20E‐01
rs141918675	4	43169480	4p13	0.01	T	A	3.00	1.94–4.69	9.74E‐07	368	4.07E‐01
**rs11726465**	4	**178378317**	**NEIL3**	**0.14**	A	C	**1.70**	1.39–2.03	**6.03E–08**	**203**	**7.88E–02**
rs770303	5	19473917	*CDH18*	0.25	A	G	1.50	1.25–1.74	5.93E‐06	241	1.70E‐01
rs111677893	6	31347069		0.35	T	C	0.70	0.55–0.78	1.37E‐06	285	9.45E‐01
**rs117495448**	7	**138359303**	***SVOPL***	**0.04**	A	G	**2.20**	1.67–3.02	**9.98E–08**	**255**	**1.85E–02**
rs113628813	11	11726915	11p15.4‐15.3	0.01	T	G	3.00	1.92–4.77	1.88E‐06	369	4.06E‐01
rs192721285	12	70339563	*MYRFL*	0.02	A	C	2.40	1.65–3.41	2.74E‐06	323	8.22E‐01
rs140649861	12	117284214	*RNFT2*	0.04	A	G	2.00	1.50–2.78	6.94E‐06	346	4.23E‐03
rs74436390	12	120184134	*CIT*	0.01	A	T	3.00	1.85–4.86	7.86E‐06	423	6.29E‐01
rs1451902	12	130248986	*TMEM132D*	0.39	C	T	1.40	1.22–1.66	6.17E‐06	287	7.04E‐01
rs142002811	13	51963277	*INTS6*	0.01	C	T	3.00	1.84–4.79	8.94E‐06	388	6.39E‐01
rs61200745	13	85238840	13q.31.1	0.13	C	A	1.60	1.32–1.97	2.72E‐06	284	5.34E‐01
rs77395534	13	100458813	*CLYBL*	0.04	G	A	2.20	1.58–2.93	1.16E‐06	278	1.74E‐01
rs35337719	13	111772219	*ARHGEF7*	0.06	T	C	1.80	1.42–2.38	4.01E‐06	330	6.43E‐01
rs7160724	14	28219361	14p12	0.48	C	T	0.70	0.59–0.81	6.00E‐06	252	6.40E‐01
rs76895919	14	56800208	*PELI2*	0.01	T	C	2.90	1.81–4.52	6.95E‐06	383	4.08E‐01
rs148414906	14	82337126	14q31.1	0.02	A	T	2.30	1.60–3.34	8.64E‐06	407	6.92E‐02
rs56069748	15	88373203	*NTRK3*	0.02	A	T	2.60	1.74–3.75	1.81E‐06	333	7.75E‐01
rs140084235	16	18024168	16p12.3	0.01	T	A	3.10	1.99–4.90	7.38E‐07	356	6.43E‐01
rs3746895	21	43162200	*RIPK4*	0.33	T	C	0.60	0.54–0.77	1.85E‐06	151	3.12E‐02
Ambidexterity											
rs150150088	1	92758007	*GLMN*	0.01	A	G	2.30	1.59–3.21	5.21E‐06	632	4.11E‐01
1:173302463:GA_G	1	173302463		0.06	R	D	1.60	1.28–1.88	7.98E‐06	526	1.00E+00
rs186743	3	3178322	*TRNT1*	0.28	A	G	1.30	1.15–1.45	8.76E‐06	540	2.70E‐01
rs75578608	3	79347280	*ROBO1*	0.03	C	G	1.80	1.38–2.28	7.85E‐06	608	1.00E+00
rs138907675	3	118725079	*IGSF11*	0.07	G	A	0.50	0.41–0.69	2.31E‐06	317	1.16E‐02
4:188281625:G_GGG	4	188281625		0.34	I	R	1.30	1.18–1.47	1.04E‐06	496	5.21E‐01
rs4476693	5	10882977	*CTNND2*	0.28	T	C	1.30	1.16–1.46	6.10E‐06	542	8.54E‐01
**rs118077080**	5	**76505576**	***PDE8B***, ***WDR41***	**0.02**	G	T	**2.10**	1.60–2.72	**6.69E–08**	**453**	**8.23E–01**
rs145242715	5	76805030	*WDR41*	0.01	A	C	2.30	1.64–3.35	3.11E‐06	677	1.00E+00
rs79041957	5	100069852	*ST8SIA4*	0.06	C	A	1.60	1.33–2.00	2.60E‐06	578	4.12E‐02
rs80114285	8	49793013	*SNAI2*	0.02	G	A	2.10	1.51–2.82	5.32E‐06	600	3.75E‐01
rs7862596	9	10407141	*PTPRD*	0.23	C	T	1.30	1.17–1.49	7.89E‐06	605	7.86E‐04
rs182637278	9	93491736	*SYK*	0.01	C	G	2.30	1.65–3.35	2.35E‐06	683	4.48E‐02
rs263422	10	8068860	*GATA3*	0.08	C	T	1.50	1.26–1.79	5.15E‐06	575	2.13E‐01
rs12606549	18	55022163	*ST8SIA3*	0.29	T	C	1.30	1.16–1.46	8.43E‐06	534	3.91E‐04

Abbreviations: HWE, Hardy‐Weinberg equilibrium.

^a^ Sample size for 80% power at *α* = .05 is based on the parameter of this population, including minor allele frequency and effect size of the markers.

**FIGURE 2:**
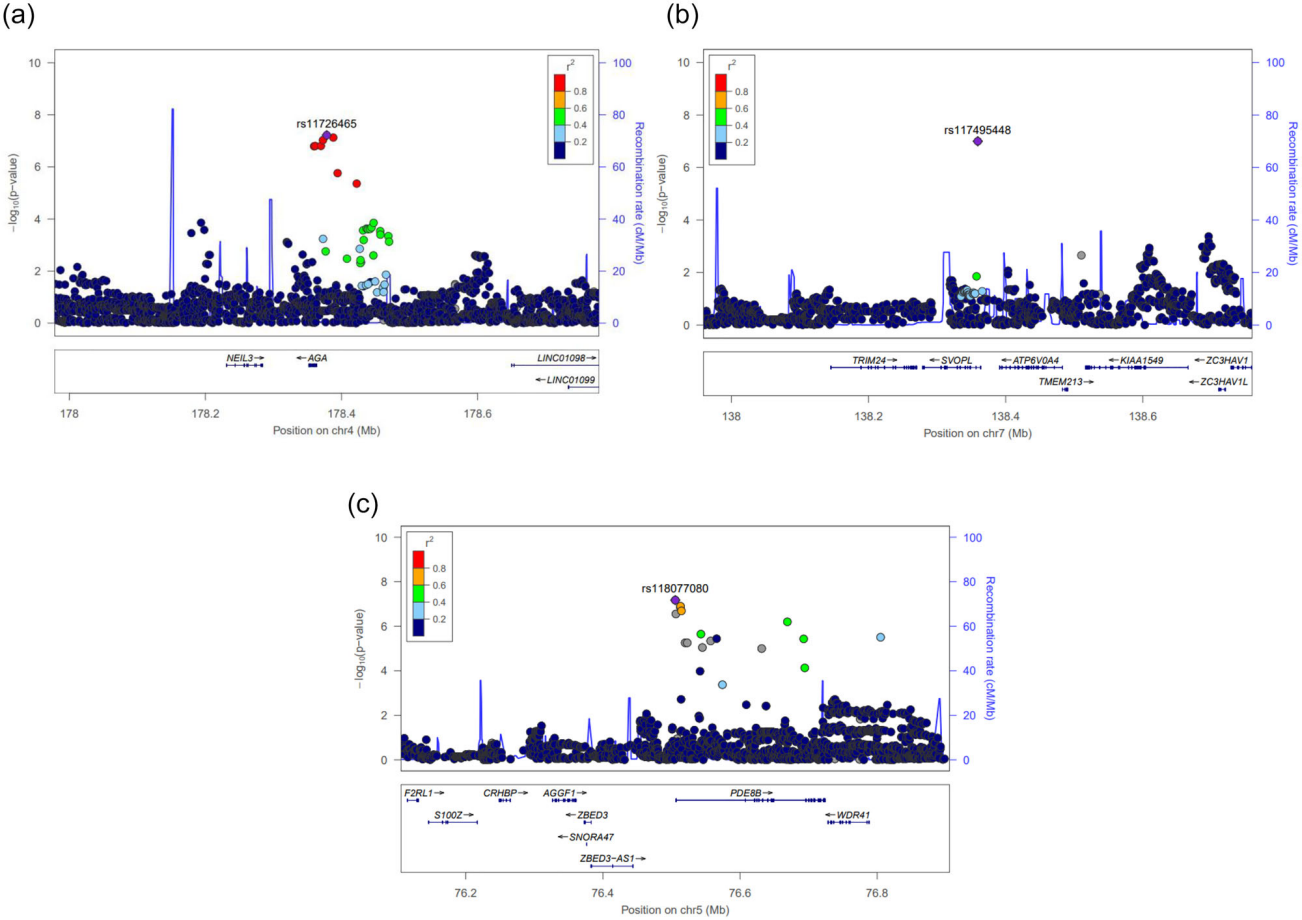
Regional association plots for lead single nucleotide polymorphisms (SNPs). (a/b) Left‐handedness and (c) ambidexterity. SNP is plotted by chromosomal position (hg19; *x*‐axis) and association with handedness from the current study (–log_10_ (p‐value); *y*‐axis).

Among the above, *rs11726465* (*NEIL3*, OR = 1.7, 95% C.I. = 1.4−2.0, *p*‐value = 6 × 10^−8^) and *rs117495448* (*SVOPL*, OR = 2.2, 95% C.I. = 1.7−3.0, *p*‐value = 9 × 10^−8^) for left‐handedness, and *rs118077080* (*PDE8B*/*WDR41*, OR = 2.1, 95% C.I. = 1.6−2.7, *p*‐value = 6 × 10^−8^) for ambidexterity nearly met the threshold for genome‐wide significance (*p*‐value < 5 × 10^−8^). The respective signal plots are shown in Figure 2.

## 4 DISCUSSION

To the best of our knowledge, this study represents the first East‐Asian GWAS for handedness to date. We used data from KoGES, a database representative of the Korean population to perform a GWAS analysis, and identified significant genetic loci associated with left‐handedness and ambidexterity; we further identified their candidate genes and hypothesized potential genetic correlations between handedness and neuropsychiatric development or traits. We also conducted association analyses with the results of the largest genetic study to date by Cuellar‐Partida et al. to identify common loci associated with handedness between Caucasians and East‐Asians.

Several studies performed worldwide have attempted to identify how handedness develops biologically and culturally; however, the underlying mechanism remains unclear (de Kovel et al., 2019; de Kovel & Francks, 2019; Michel, 2021; Papadatou‐Pastou et al., 2020). Moreover, Asian populations have been relatively under‐represented in these studies (Zheng et al., 2020). Twin and familial studies have shown that approximately 25% of the variance in handedness can be attributed to genetics (de Kovel et al., 2019; Zheng et al., 2020). However, only a fraction of this percentage can be attributed to specific genes, and relevant large‐scale studies are ongoing to uncover more of these. Genetic correlations identified to date implicate high polygenicity and shared biology between handedness, cerebral asymmetries, neurodevelopment/plasticity, neurodegenerative processes, and neuropsychiatric diseases (Cuellar‐Partida et al., 2021; Kong et al., 2021; Paracchini, 2021). In addition, left‐handedness and ambidexterity have been suggested to be influenced by different genetic mechanisms (Cuellar‐Partida et al., 2021).

Among the 28 left‐handedness loci, two (*NEIL3* and *SVOPL*) showed near genome‐wide significance. *NEIL3*, an important gene related to DNA repair, has been widely implicated in diverse brain functions (Hildrestrand et al., 2009; Kunath et al., 2021; Regnell et al., 2012). Recently, *NEIL3* has been identified to enable stable neural representation of space by shaping CA1 transcription in mouse hippocampal neurons, with NEIL3^−/−^ mice showing impaired spatial performance (Kunath et al., 2021). Other studies have also shown an important role of *NEIL3* in fetal neurogenesis (Hildrestrand et al., 2009), as well as maintenance of adult neurogenesis in the hippocampus, thus implicating associations with neurodegenerative disease (Regnell et al., 2012). Further, composite substance dependence phenotypes (Wetherill et al., 2014) and impulsivity (Ehlers et al., 2016) associations have been reported. These findings are interesting because handedness (as well as temporal lobe epilepsy) has been identified to affect right‐to‐left amygdalar and hippocampal volume ratios (Szabo et al., 2001).

*SVOPL* is a paralog of the *SVOP* gene that encodes synaptic vesicle 2 (SV2)‐related proteins expressed in neurons, and is expressed in all brain regions while appearing earlier than SV2 in development (Yao et al., 2013). *SVOPL* has been found to be significant in Parkinson's disease by large‐scale whole‐exome sequencing (Jansen et al., 2017), potentially deleterious in autosomal‐dominant lateral temporal epilepsy (Dazzo et al., 2015), and is associated with a super‐variant associated with brain connectivity (Li et al., 2021). Further, it has been found to be associated as a non‐word repetition marker in specific language impairment risk (Luciano et al., 2013). Although accumulating evidence suggests a genetic relationship in variants associated with handedness (especially left‐handedness), cerebral asymmetry, and neuropsychiatric/developmental disorders such as dyslexia, Parkinson's disease, and schizophrenia, these remain to be clearly identified (Brandler & Paracchini, 2014; Wiberg et al., 2019).

Of the 15 ambidexterity loci, *PDE8B* showed the highest, near genome‐wide significance. *PDE8B* plays a major role in the breakdown of cyclic AMP, a key messenger in dopamine signaling (Bollen & Prickaerts, 2012). *PDE8B* expression analysis in humans has shown the highest levels in the striatum, hippocampal formation, cerebellum, and cortex (Pérez‐Torres et al., 2003); hippocampus‐dependent brain functions such as spatial pattern recognition and contextual fear are known to be regulated by cAMP signaling; further, striatal regulation of cAMP downstream from dopamine pathways plays a major role in numerous aspects of motor function as well as motivated behavior and reward learning. Accordingly, *PDE8B* has been implicated in autosomal‐dominant striatal degeneration (Appenzeller et al., 2010), with higher levels observed in Alzheimer's patients (Pérez‐Torres et al., 2003); in agreement with these findings, PDE8B KO mice demonstrate enhanced motor performance as well as alleviated motor coordination decay (Tsai et al., 2012). Along with *PDE8B*, the adjacently located *WDR41* is associated with dopamine signaling and development as well as autophagy in neurons; it is also suspected to be involved in ALS/FTLD (Budini et al., 2017; Goodier et al., 2020; Sullivan et al., 2016). These two genes were identified to have peak associations in a study on caudate volume, which suggested relevance to common conditions affecting the caudate (Stein et al., 2011). We consider this finding as significant because the basal ganglia including the caudate is a brain structure involved in many common neurological and psychiatric diseases as it regulates the dopaminergic system; cerebral asymmetries involving this region have been previously found to have associations with such disorders as well as handedness (Jang et al., 2017; Peterson et al., 1993). Additionally, functional nigrostriatal dopamine system asymmetry has been identified in motor lateralization using fluorodopa‐PET (de la Fuente‐Fernandez et al., 2000).

Other genes near the variant also showed involvement in brain development (*ZBED3*) (Ruan et al., 2021), in GABAergic interneurons in the prefrontal cortex (Ketchesin et al., 2017), hippocampal abnormalities (Ensink et al., 2021), depression, and other neuropsychiatric disorders such as substance abuse (*CRHBP*) (Curley et al., 2021; Kalin, 2018).

Among the 28 suggestively significant left‐handedness variants, we could identify 20 genes proximal to the loci; of these, 13 are related to neurological pathways such as synapses, neurons, or neuronal signaling (*AGA* (Chen et al., 2021; Saarela et al., 2004), *SVOPL* (Yao et al., 2013), *SGIP1* (Hajkova et al., 2016), *KLB* (Jackson et al., 2019), *NTRK3* (Ito et al., 2020), *CX3CR1* (Pawelec et al., 2020), *CACNA1E* (Carvill, 2019), *ARHGEF7* (Lopez Tobon et al., 2018), *TRIB2* (Dobens et al., 2021), *CDH18* (Bai et al., 2018), *TAFA1* (Sarver et al., 2021), *PELI2* (Dai et al., 2019), and *CIT* (Ahmed et al., 2011)). Further, four genes are correlated with neuropsychiatric traits such as behavioral disorders, panic disorders, or Alzheimer's disease (*MYRFL* (Anney et al., 2008), *TNEN132* (Naik et al., 2018), *RNFT2* (Kamboh et al., 2012), and *PLA2G4A* (Sarkar et al., 2020)). Similarly, among the 15 suggestively significant ambidexterity‐associated variants, we located 13 proximal genes. Of these, eight genes are related to cell signaling or neurons (*PDE8B* (Pearse et al., 2004; Fan et al., 2018), *ST8SIA4* (Berger et al., 2016), *WDR41* (Goodier et al., 2020), *GATA3* (Andrzejczuk et al., 2018), *SNAI2* (Wei et al., 2020), *CTNND2* (Turner et al., 2015), *ROBO1* (G. Wang et al., 2013), and *PTPRD* (Tomita et al., 2020)), and three are associated with brain development or neurodevelopmental, degenerative disorders (*IGSF11* (Higashine et al., 2018), *SYK* (Angibaud et al., 2011; Nazarian et al., 2019), and *TRNT1* (Chakraborty et al., 2014)).

We also performed association analyses1164 with the findings of Cuellar‐Partida et al. that are based on Caucasians and could replicate two variants in the Korean population. In left‐handedness and ambidexterity, *rs7132513* and *rs2040498*, respectively, showed common significance between the two study populations (*p*‐value ≤ .05). *ANKS1B* (*rs7132513*) encodes a protein predominantly expressed in the brain and testis, which interacts with amyloid beta protein precursor, and may play a role in normal neurodevelopment as well as the pathogenesis of Alzheimer's disease (Carbonell et al., 2019). *ANKIB1* (*rs2040498*) is a protein‐coding gene predicted to be involved in ubiquitin‐associated activity and has been identified in angiokeratoma corporis diffusum with arteriovenous fistulas and autism spectrum disorder (L.‐S. Wang et al., 2010). Further, an important gene is *RNF19A*, which encodes the protein E3 ubiquitin ligase localized to Lewy bodies, which may be involved in ALS and Parkinson's disease (Park et al., 2015).

This study has several merits. To the best of our knowledge, it is the first GWAS on East‐Asians to date, and the cohort used for analysis is a well‐known large database representative of the Korean population. Two common variants were replicated from the largest handedness GWAS to date in a different ethnicity; both variants were correlated with genes involved in neurological processes. Further, owing to the unique East‐Asian culture of disregarding left‐handedness and strongly enforcing right‐handedness, the identified left‐handedness associated variants may have stronger predictive power than others. Our database included a large middle‐aged population between 40−60 years of age, which was raised in a society in which left‐handedness was taboo (only 2% of Koreans in their 60s identified as left‐handed). The participants who maintained left‐handedness despite environmental coercion may have been largely affected by the genetic variants identified in this study. The variants and candidate genes identified in this study potentially implicate novel associations or reinforce known relations between handedness and neurologic processes and conditions. However, it is not clear whether these relations between handedness and neuropsychiatric traits are directly, independently associated based on our analysis; further studies are warranted to explore the exact nature of these associations based on our findings.

However, this study has limitations. Although some variants nearly met the threshold for genome‐wide significance (*p*‐value < 5 × 10^−8^), most showed suggestive significance (5 × 10^−8^ ≤ *p*‐value < 1 × 10^−5^). Multiple correction may have affected the results; although the yield of replication findings and a significant meta‐analysis support our findings, further validation and international as well as domestic replication studies are warranted. Additionally, the inclusion of association loci based on European ancestry (and replication on such dataset) is another limitation, which is due to the current lack of Asian studies. Lastly, in this study, we observed associations with *p*‐value < .05 based on general GWAS and replication reports (Gonzalez et al., 2016), but from a statistical aspect, said *p*‐value would not be significant by Bonferroni correction.

In conclusion, we present the first study to identify genetic variants associated with handedness in Koreans. We identified 28 and 15 variants associated with left‐handedness and ambidexterity, respectively (*p*‐value ≤ 10^−5^); of these, two and one variants associated with left‐handedness and ambidexterity showed genome‐wide significance, respectively. Further, one variant each associated with left‐handedness and ambidexterity was replicated from the results of a large Western study (*p*‐value < .05). Our results showed limited genetic similarity between left‐handedness and ambidexterity in line with previous studies, again suggesting an independent genetic architecture. Further, both handedness‐associated variants showed potential relations with genes involved in neurodevelopment or neuropsychiatric traits, suggesting laterality as polygenic and the result of complex neurological genetic pathways as implicated in previous studies. Further studies with larger databases and different ethnicities are warranted; overall, this study hopes to provide a stepping stone for future research into human behavior and brain development.

## CONFLICT OF INTEREST STATEMENT

The authors declare no conflicts of interest.

### PEER REVIEW

The peer review history for this article is available at https://publons.com/publon/10.1002/brb3.3121.

## Supporting information

Supplementary Figure 1. Quantile‐quantile plots with genomic inflation values. a) Right‐handedness–Left‐handedness and b) Right‐handedness–Ambidexterity

Supplementary Table 1. Left‐handedness associated GWAS results

Supplementary Table 2. Ambidexterity associated GWAS results

Supplementary Table 3. Replication GWAS original result

Supplementary Table 4. (B) Meta‐analysis for ambidexterity with replication dataset

## Data Availability

The data that support the findings of this study are available from the corresponding authors upon reasonable request.
