# Supplementary figures and images for: Genome‐wide association and replication studies for handedness in a Korean community‐based cohort
Source: Brain Behav. 2023 Jun 20;13(9):e3121. doi: 10.1002/brb3.3121 (PMC10498080; doi:10.1002/brb3.3121)

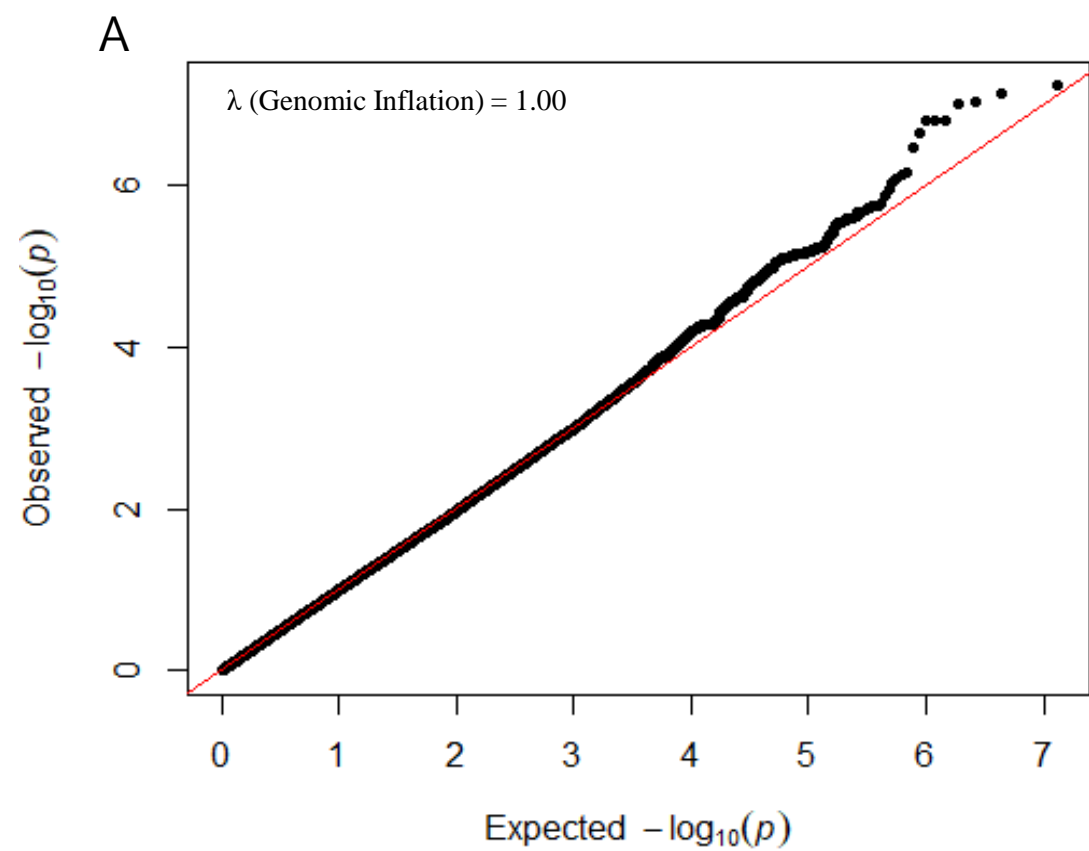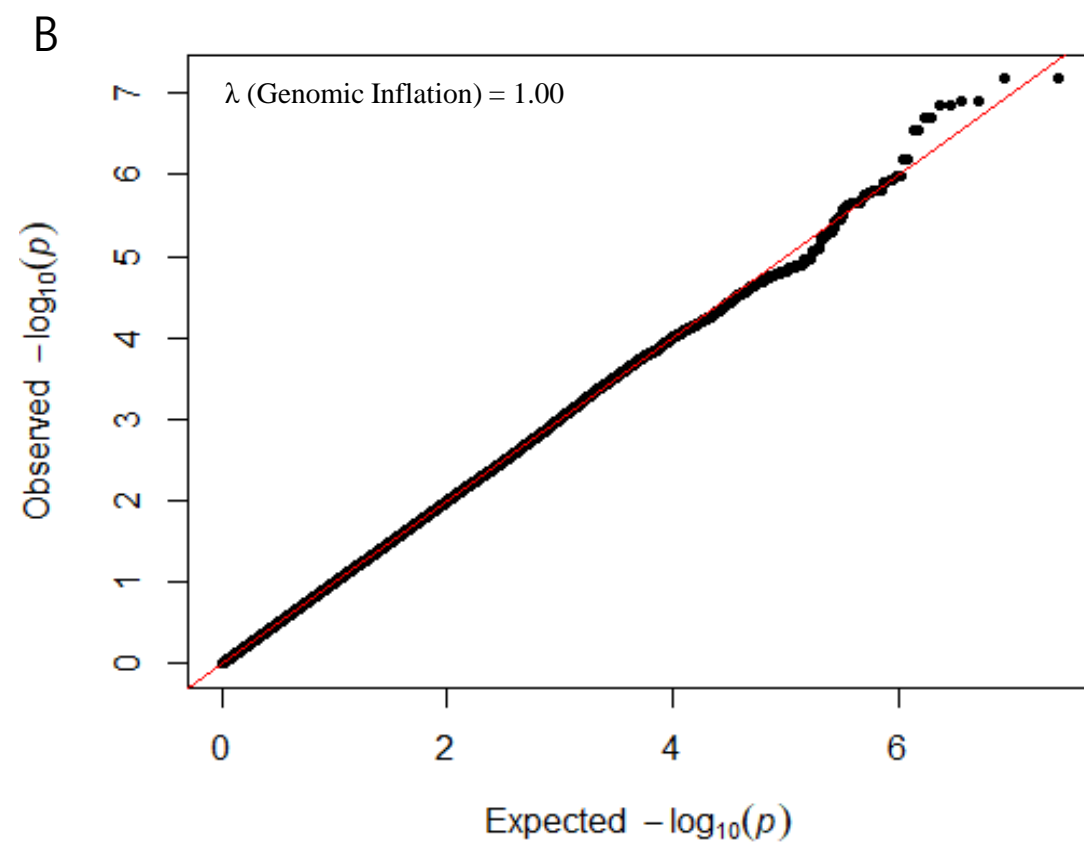

Supplement: Supplementary file 1 — Supplementary Figure 1. Quantile‐quantile plots with genomic inflation values. a) Right‐handedness–Left‐handedness and b) Right‐handedness–Ambidexterity [file BRB3-13-e3121-s001.pdf]
